# Multiple Autoimmune-Associated Variants Confer Decreased IL-2R Signaling in CD4^+^CD25^hi^ T Cells of Type 1 Diabetic and Multiple Sclerosis Patients

**DOI:** 10.1371/journal.pone.0083811

**Published:** 2013-12-23

**Authors:** Karen Cerosaletti, Anya Schneider, Katharine Schwedhelm, Ian Frank, Megan Tatum, Shan Wei, Elizabeth Whalen, Carla Greenbaum, Mariko Kita, Jane Buckner, S. Alice Long

**Affiliations:** 1 Translational Research, Benaroya Research Institute at Virginia Mason, Seattle, Washington, United States of America; 2 Bioinformatics, Benaroya Research Institute at Virginia Mason, Seattle, Washington, United States of America; 3 Diabetes Research, Benaroya Research Institute at Virginia Mason, Seattle, Washington, United States of America; National Institutes of Health, United States of America

## Abstract

IL-2 receptor (IL-2R) signaling is essential for optimal stability and function of CD4^+^CD25^hi^FOXP3^+^ regulatory T cells (Treg); a cell type that plays an integral role in maintaining tolerance. Thus, we hypothesized that decreased response to IL-2 may be a common phenotype of subjects who have autoimmune diseases associated with variants in the *IL2RA* locus, including T1D and MS, particularly in cells expressing the high affinity IL-2R alpha chain (IL-2RA or CD25). To examine this question we used phosphorylation of STAT5 (pSTAT5) as a downstream measure of IL-2R signaling, and found a decreased response to IL-2 in CD4^+^CD25^hi^ T cells of T1D and MS, but not SLE patients. Since the *IL2RA*rs2104286 haplotype is associated with T1D and MS, we measured pSTAT5 in controls carrying the rs2104286 risk haplotype to test whether this variant contributed to reduced IL-2 responsiveness. Consistent with this, we found decreased pSTAT5 in subjects carrying the rs2104286 risk haplotype. Reduced IL-2R signaling did not result from lower CD25 expression on CD25^hi^ cells; instead we detected increased CD25 expression on naive Treg from controls carrying the rs2104286 risk haplotype, and subjects with T1D and MS. However the rs2104286 risk haplotype correlated with increased soluble IL-2RA levels, suggesting that shedding of the IL-2R may account in part for the reduced IL-2R signaling associated with the rs2104286 risk haplotype. In addition to risk variants in *IL2RA,* we found that the T1D-associated risk variant of *PTPN2*rs1893217 independently contributed to diminished IL-2R signaling. However, even when holding genotype constant at *IL2RA* and *PTPN2*, we still observed a significant signaling defect in T1D and MS patients. Together, these data suggest that multiple mechanisms converge in disease leading to decreased response to IL-2, a phenotype that may eventually lead to loss of tolerance and autoimmunity.

## Introduction

The cytokine IL-2 is essential for T cell homeostasis. Activated effector T cells (Teff) produce IL-2 and transiently up-regulate the high affinity IL-2RA (CD25) upon activation, enabling them to respond optimally to IL-2 following antigen encounter. Expression of CD25 on Teff is then reduced through negative feed-back loops as the cells come to rest. Enzymes can also cleave CD25 from the surface of T cells upon activation resulting in soluble IL-2RA (sIL-2RA) that can be detected in the serum [1]–[3]. In contrast to Teff, regulatory T cells (Treg) which are essential for suppressing autoimmunity do not produce IL-2 themselves, but paradoxically are highly dependent on IL-2 for their survival and function [4]. Treg constitutively express high levels of CD25 and therefore are highly sensitive to even low doses of IL-2. IL-2R signaling in both Teff and Treg is mediated by a complex of CD25, the IL-2R beta chain (IL-2RB or CD122) and the common gamma chain. Alternatively, the IL-2RB and common gamma chain can serve as a low affinity IL-2R. Upon cytokine binding the IL-2R, a series of sequential phosphorylation events are initiated from the beta and gamma chains including phosphorylation of JAK1, JAK3 and Shc proteins resulting in transcriptional activation of cytokine-targeted genes, including the STAT5-dependent Treg transcription factor FOXP3 and CD25 itself [5].

Impairment of IL-2/IL-2R signaling has striking consequences on the development of tolerance to self-antigens. This is demonstrated most dramatically in knock-out mice in which a deficiency of *Il2*, *Il2ra,* or *Il2rb* leads to early death due to severe autoimmunity [6]–[8]. In humans, deficiency of IL-2RA can result in autoimmunity [9], [10]. Consistent with this, we have observed decreased response to IL-2 in CD25^+^ and memory T cells of T1D subjects [11]. However, it is not clear whether this deficit is common to all autoimmune settings.

The importance of the IL-2R pathway in maintaining tolerance is also revealed by genome wide association studies (GWAS) that have associated multiple variants in the IL-2/IL-2R signaling pathway with susceptibility for type 1 diabetes (T1D), multiple sclerosis (MS), rheumatoid arthritis, Crohn’s disease, Grave’s disease, generalized vitiligo, and alopecia areata. Specifically, autoimmune variants have been identified in the *IL2RA* gene [12]–[20], as well as in *IL2* itself [12], [19], [20], *IL2RB* [12], and the protein tyrosine phosphatase N2 (*PTPN2*)[12], [20], a phosphatase in multiple signaling pathways including the IL-2R signaling pathway [21]–[23]. Amongst these genes, *IL2RA* is unique to the IL-2R pathway, whereas the other genes function in other cytokine pathways in addition to IL-2.

Genetic association of the *IL2RA* locus with autoimmunity is complex. Three haplotypes that are tagged by three single nucleotide polymorphisms (SNPs) have been defined in the *IL2RA* locus: the rs12722495 (previously rs41295061) protective haplotype that is only associated with T1D (OR = 0.62, p = 6.43×10^−25^), the rs2104286 protective haplotype that is associated with MS (OR = 0.85, p = 6.27×10^−7^), T1D (OR = 0.80, p = 1.27×10^−13^) and RA (OR = 0.76, p = 4.9×10^−5^), and the rs11594656 haplotype that is associated with protection from T1D (OR = 0.87, p = 3.37×10^−6^) but risk for MS (OR = 1.17, p = 7.67×10^−4^)[16], [24], [25]. Other SNPs in the *IL2RA* locus are associated with Crohn’s disease, Grave’s disease, vitiligo, and alopecia areata [14], [15], [17], [19], [26]. All of the associated variants are non-coding, falling upstream of the *IL2RA* gene or in intron 1, and despite being in high linkage disequilibrium (D’ =  1.0); many of these SNPs have low R^2^ values.

Understanding if altered IL-2R signaling is a phenotype common to multiple autoimmune diseases and how this relates to the genetic heterogeneity at the *IL2RA* locus is important for elucidating mechanisms common or unique to associated autoimmune diseases. Here, we report reduced IL-2 responsiveness in T1D and MS, and define multiple phenotypes that correlate with the *IL2RA*rs2104286 risk haplotype that is associated with these diseases. We also demonstrate that additional risk alleles in the IL-2R signaling pathway contribute independently to decreased IL-2 responsiveness, collectively impacting tolerance.

## Materials and Methods

### Human Subjects

PBMC were derived from subjects participating in studies under the auspices of the BRI-JDRF Center for Translational Research registry. This study was approved by the Benaroya Research Institute IRB. Written informed consent was obtained from all subjects according to IRB approved protocols at Benaroya Research Institute, Seattle. Subject demographics, genotype and clinical characteristics are described in Table 1. Control participants were selected based on lack of personal or family history of autoimmunity or asthma. Samples for this study were obtained from adult control subjects and subjects diagnosed with T1D, MS and SLE. For some experiments, samples were provided by the Genotype and Phenotype Registry, a service of the Tissue Donation Program at The Feinstein Institute for Medical Research, Manhasset, New York, USA. Samples for all experiments were provided to the investigator by the BRI clinical core in a blinded manner. This enables prospective selection of subjects for individual experiments based on demographic, genetic and clinical characteristics without the experimenter’s prior knowledge of disease state, genotype or subject information. Control and disease subjects or risk and non-risk subjects were included in each batch received from the clinical core to avoid batch effects on experimental data.

**Table 1:** Characteristics of cohorts.

Cohort	Healthy Control	T1D	MS^b^	SLE^c^
n	181	48	55	20
age^a^	36±16	33±12	37±14	48±14
	(18–68)	(18–74)	(22–63)	(25–79)
% Female	57	42.4	83.9	70.0
% Caucasian	86.6	94.7	98.4	95.0
% non-Hispanic	97.8	96.4	100	80
Disease status	NA^d^	200 – 22,488 days	94% RRMS	93% arthritis
		since diagnosis	3% PPMS	87% skin
		(ave: 6537, SD 5264)	3% SPMS	31% renal
				25% pleurisy
Current Immuno-	None	None	55% untreated^e^	63% HCQ
modulatory			40% Tysabri	37% Prednisone
medications at			3% Copaxone	16% MMF
time of draw			2% Interferons	5% methotrexate
				5% azathioprine
				5% belimumab
*IL2RA*rs2104286^f^				
A/A	106 (0.586)	24 (0.500)	35 (0.636)	ND^g^
A/G	68 (0.376)	16 (0.333)	13 (0.236)	ND
G/G	6 (0.033)	8 (0.167)	7 (0.127)	ND
Freq A	0.778	0.667	0.755	ND
Freq G	0.222	0.333	0.245	ND

^a^ average ± SD years (range).

^a^ ave ± SD (range).

^b^ Relapsing Remitting, Primary Progressive and Secondary Progressive Multiple Sclerosis.

^c^ SLE disease characteristics were determined by organ involvement at any point in the subjects disease course; HCQ- hydroxychloroquine; MMF- mycophenylate mofetil.

^d^ Not Applicable.

^e^ untreated includes subjects not on immune modulatory drugs >3 months prior to blood draw

^f^ n (frequency).

^g^ Not Determined.

### Genotyping

*IL2RA*rs12722495, *IL2RA*rs2104286, *IL2RA*rs11594656, and *PTPN2*rs1893217 were genotyped using fluorescently labeled MGB-Eclipse system (Epoch Biosciences). The genotyping assay was performed using 10ng of genomic DNA, 0.38U JumpStart™ Taq DNA polymerase (Sigma-Aldrich), primers and probes in a 5 µl reaction volume according to the manufacturer’s protocol. PCR was performed and analyzed on an ABI HT7900.The *IL2RA*rs2104286 haplotype allows variation at rs2104286 while holding rs12722495 constant for the A/A genotype and rs11594656 constant for the T/T genotype as described by Dendrou et.al [25].

### Flow cytometric analysis for phosphorylated STAT5

BD Phosphoflow staining was performed as per manufacturer’s instructions. In brief, cells were activated with different concentrations of IL-2 or IL-15 for 10 and/or 20min, fixed with Phosflow Buffer I and permeabilized using BD Phosflow Buffer III prior to staining with Alexa647 anti-pSTAT5(Y694), PerCP CD4 and PE CD25 (clone M-A251). For some experiments, cells were co-stained with FITC anti-CD45RO. Initial experiments were performed using freshly isolated PBMC. As shown previously[25], similar results were obtained with thawed PBMC. All pSTAT5 data shown in this manuscript are from previously frozen PBMC. Data were acquired using a FACS Calibur and analyzed using FloJo software. Since CD4^+^CD25^hi^ T cells are known to be sensitive to the freeze/thaw process and fix/perm staining protocols, samples where less than 1% of CD25^hi^ T cells or <500 events were detected were excluded from results. pSTAT5(Y694) mean fluorescence intensity (MFI) data were normalized between experiments by determining the MFI fold increase (geometric MFI of the positive population ÷ geometric MFI of the negative control) as described previously [27]

### Flow cytometric analysis for surface and nuclear markers

Thawed PBMC were surface stained with V500 CD4 and PE CD25 prior to fixation and permeabilization using BioLegend FOXP3 Buffer sets. Permeabilized cells were then stained with Alexa647 anti-FOXP3 and Alexa488 anti-helios antibodies. Invitrogen 8 peak beads were used to normalize flow cytometry settings between experiments by adjusting voltage settings to reach a standard MFI.

### sIL-2RA ELISA

sIL-2RA serum levels were determined using Luminex plates as per manufacturer’s instructions. Values were log10 transformed to achieve a normal distribution [16], [24].

### Statistics

Based on preliminary data, we performed all experiments using the number of samples we calculated were required to achieve 80% power to detect a 10% difference between groups. The 10% difference was selected as a biologically relevant difference based on published work [11], [25] and a value that was greater than twice the % CV for repeat measures on the same sample. For observed data reported in this manuscript, all experiments with multiple group comparisons were first analyzed using a Kruskal-Wallis non-parametric ANOVA test to determine whether any group differed from the others. Individual pairings were analyzed using a Mann-Whitney test to determine significance. Results of Mann-Whitney testing including 95% confidence intervals for the difference between the medians are reported in Table S1. False discovery rate was determined using the Benjamini Hochberg procedure. Permutation testing was performed on all pair-wise comparisons. Outliers were identified using both control charts and the Grubb’s test. All outliers are noted in the Figure Legends. For analysis of multiple variables, linear regression was performed as noted in the figure legends. Interaction of *IL2RA* and *PTPN2* genotype on IL-2 responsiveness was assessed using PLINK (http://pngu.mgh.harvard.edu/~purcell/plink/) with an additive SNP genotype model for *IL2RA* genotype using the “linear” command, including *PTPN2* genotype as a covariate with and without the “interaction” command. Association testing was performed using a Chi-square test and interaction of variants was modeled using the R software package. Comparisons required a p-value of <0.05 for the data to be considered significantly different.

## Results

### T1D and MS, but not SLE subjects display decreased response to IL-2 in CD4^+^CD25^hi^ T cells

**Figure 1:**
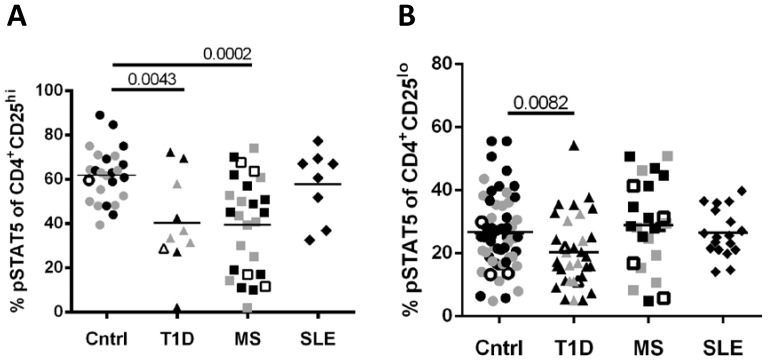
Decreased response to IL-2 in CD4^+^CD25^+^ T cells is a phenotype common to T1D and MS, but not SLE subjects. PBMC were thawed, rested and stimulated with media alone or IL-2 (100 IU/ml) prior to fixation, permeabilization and staining with CD4, CD25, CD45RO and pSTAT5. (A) The frequency of pSTAT5^+^ cells of CD25^hi^ cells was determined by comparing media to 25 IU IL-2 stimulation for 20min. (B) In an independent cohort, the frequency of pSTAT5^+^ cells of CD25^lo^ cells was determined by comparing media to 100 IU IL-2 stimulation for 10min. In all experiments, patients and controls were prospectively matched with controls for age and ethnicity. Groups in (A) and (B) differed by multiple parameter testing using a Kruskal-Wallis test with p-values of 0.0008 and 0.0198, respectively. Statistical significance for individual pairings using a Mann-Whitney test are shown. Differences shown in (A) were verified with permutation testing where control vs T1D and control vs MS remained significant (<0.001). For (B), after permutation testing the control vs T1D (0.005), T1D vs MS (0.03) and T1D vs SLE (0.01) pairings remained significant. There was one outlier in the T1D cohort in (B). By ANOVA, age, gender, race, disease status or immune treatment did not significantly contribute to differences in % pSTAT5 for any cohort (therapy and disease status analyses are shown in Figure S3). The mean pSTAT5 response is indicated for each group. *IL2RA*rs2104286 genotype is noted by solid black (A/A), solid grey (A/G) and open (G/G) symbols.

We have previously observed reduced response to IL-2 in T cells from subjects with T1D [11]. One downstream functional measure of IL-2/IL-2R signaling is phosphorylation of STAT5 (pSTAT5) in response to IL-2 stimulation. To determine if reduced IL-2 responsiveness is a common feature of autoimmunity, we measured pSTAT5 in response to IL-2 in CD4^+^CD25^hi^ T cells of T1D, MS and SLE patients. Both T1D and MS subjects displayed significantly reduced response to IL-2 in the CD25^hi^ populations, whereas SLE subjects did not differ from controls (Figure 1A). Consistent with previous studies, we found decreased response to IL-2 in the CD25^lo^ population of T1D subjects ([11] and Figure 1B), although we did not find this same decrease in the CD25^lo^ cells of MS or SLE patients. By ANOVA, age, gender, race, disease status or immune treatment did not significantly contribute to differences in the percentage of pSTAT5 for any cohort. Thus, a common feature of T1D and MS is a reduced response to IL-2 in CD4^+^CD25^hi^ T cells.

### The IL2RArs2104286 risk haplotype correlates with decreased IL-2R signaling in CD25^hi^ T cells from control subjects

The *IL2RA*rs2104286 risk haplotype is associated with T1D and MS [13], [16], [18], [24], but not SLE [28], [29], reflecting the response to IL-2 we observed in Figure 1. Thus, we asked whether genetic susceptibility contributes to this disease-specific phenotype. The observed reduced response to IL-2 in the CD25^hi^ cells of the MS and T1D cohorts, was not due to the percentage of subjects carrying the risk allele (control 59%, T1D 50% MS 64% A/A genotype: p = 0.1393, chi-square test Table I). There was also no significant difference in IL-2 response within each disease group when stratified by rs2104286 haplotype (T1D p = 0.2122; MS p = 0.1209).

**Figure 2:**
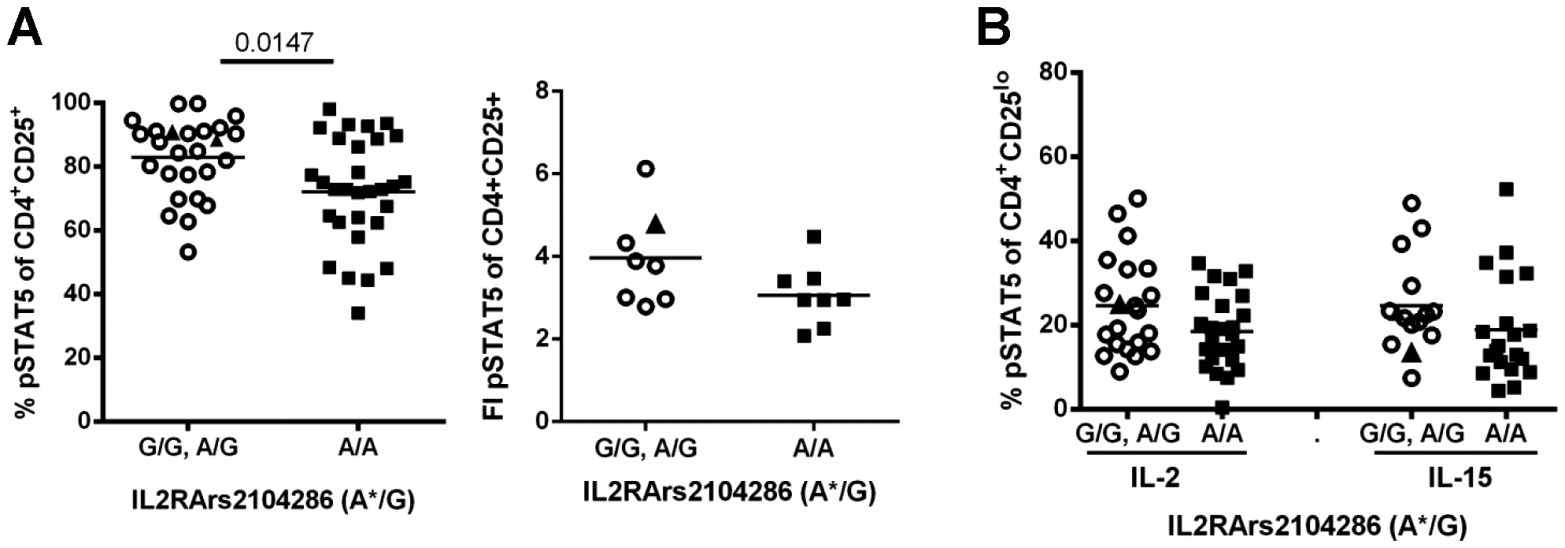
Decreased response to IL-2 in CD4^+^CD25^hi^ T cells correlates with an *IL2RA* risk haplotype associated with T1D and MS. PBMC were thawed and stained as in Figure 1. (A) The frequency and MFI fold increase of pSTAT5^+^ cells of CD25^+^ gated cells was determined by comparing media to IL-2 stimulated genotyped control samples. (B) The frequency of pSTAT5^+^ cells of CD25^lo^ was determined following stimulation of genotyped controls with IL-2 or IL-15 (200pg/ml) for 10min. The mean pSTAT5 response is indicated for each group and subjects with *IL2RA*rs2104286 genotypes G/G (▴), A/G (○) and A/A (▪) are differentiated by symbol shapes. Statistical significance was determined using a Mann Whitney test. All controls tested were Caucasian with no significant difference in age or gender between genotypes. *denotes the risk haplotype determined by looking at variation at the rs21042856 allele while holding the rs12722495 allele constant at T/T and the rs11594656 allele constant at T/T.

In order to discern the genetic contribution of rs2104286 in the absence of disease effects, we assessed pSTAT5 in response to IL-2 in healthy control subjects genotyped for the *IL2RA*rs2104286 haplotype. Variation in the rs2104286 haplotype was determined by holding the rs12722495 allele constant at A/A and the rs11594656 allele constant at T/T while looking at variation at rs2104286 as described by Dendrou et.al. [25]. The frequency and MFI of pSTAT5 in CD4^+^CD25^hi^ T cells (Figure 2A) was significantly lower in control subjects homozygous for the *IL2RA*rs2104286 risk haplotype compared to subjects carrying the A/G genotype at rs2104286. Similar results were found in a smaller but independent cohort (Figure S1).This decrease was also observed in CD45RO^+^ memory T cells, a subset containing the majority of CD25^hi^ cells, but not in naïve T cells (data not shown). We next determined if this phenotype was specific for expression of CD25, since other cytokine receptors use the gamma chain, including the IL-15R which utilizes both the common gamma chain and IL-2Rβ, but has a unique IL-15RA chain that confers specificity [30]. Although there was a trend, a significant decrease in pSTAT5 was not observed in CD25^lo^ cells stimulated with IL-2 or IL-15 (Figure 2B), cell subsets and stimuli that do not depend on CD25 expression. Thus, responses to IL-2 are decreased in CD25^hi^ T cells of control subjects homozygous for the *IL2RA*rs2104286 risk haplotype and this defect was only observed in cells expressing the IL-2RA chain

### Subjects homozygous for the IL2RArs2104286 risk haplotype with reduced IL-2 responsiveness do not display decreased CD25 expression on T cells

**Figure 3:**
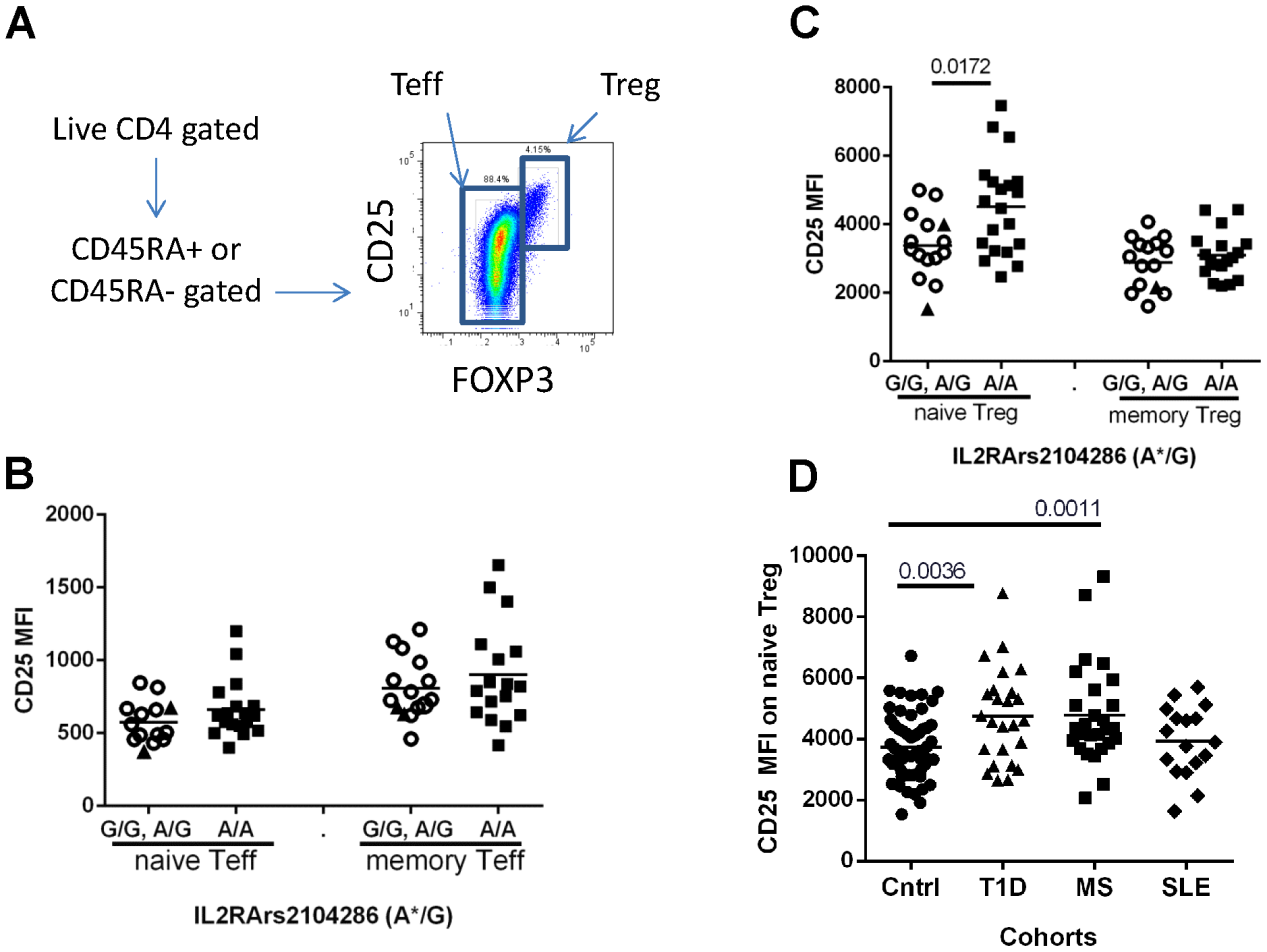
Increased expression of CD25 on naïve Treg is common to controls carrying the *IL2RA*rs2104286 risk haplotype and MS and T1D subjects. PBMC were thawed and stained for CD4, CD25, FOXP3, helios and CD45RA as described in materials and methods. (A) Representative gating of CD4^+^ T cells on FOXP3^−^ Teff and FOXP3^+^ Treg. CD25 MFI was measured on CD45RA^+^ naïve and CD45RA^−^ memory Teff (B) and Treg (C) populations of controls and (D) autoimmune subjects. The mean pSTAT5 response is indicated for each group. For (B) and (C), all controls tested were Caucasian with no significant difference in age or gender between *IL2RA*rs2104286 genotypes. G/G (▴), A/G (○) and A/A (▪) subjects are differentiated by symbol shapes. In (D), patients and controls were prospectively matched with controls for age and ethnicity. In (B-C), statistical significance between pairings was determined using a student’s t- test after assessing normality of the data as determined using the Shapiro-Wilk test. P-values were then adjusted using the Benjamini Hochberg false discovery rate procedure. Adjusted p values where significant are shown. Groups in (D) differed by multiple parameter testing using a Kruskal-Wallis test (0.0085). Statistical significance for individual pairings using a Mann-Whitney test is shown. Differences were verified with permutation testing where control vs T1D (0.015) and control vs MS (0.01) remained significant. There were two outliers in the MS cohort. *denotes the risk haplotype determined by looking at variation at the *IL2RA*rs21042856 allele while holding the rs12722495 allele constant at T/T and the rs11594656 allele constant at T/T. By ANOVA, age, gender, race, disease status or immune treatment did not significantly contribute to differences in CD25 MFI for any cohort. *IL2RA*rs2104286 genotype is noted by solid black (A/A), solid grey (A/G) and open (G/G) symbols.

Decreased response to IL-2 may be caused by lower expression of the IL-2R. Thus, we measured the surface expression of CD25 by flow cytometry on different CD4^+^ T cell subsets including naïve and memory subsets of Treg and Teff cells as determined by CD45RA and FOXP3 expression (Figure 3A). Interestingly, we did not detect decreased CD25 expression on naïve or memory Teff despite these populations displaying decreased IL-2 responsiveness (Figure 3B). Instead, we found a significant increase in CD25 expression on naïve Treg from subjects homozygous for the rs2104286 risk haplotype (Figure 3C). Moreover, this phenotype was also observed in T1D and MS subjects (Figure 3D). The observed increase in CD25 expression in the naïve Treg population of MS and T1D patients was not due to the percentage of subjects carrying the risk allele and there was no significant difference in CD25 MFI within each disease group when stratified by rs2104286 haplotype. Thus, the decreased response to IL-2 in CD25^hi^ cells observed with the rs2104286 risk haplotype in controls, as well as in T cells from subjects with T1D and MS, is not solely a result of reduced surface expression of IL-2RA.

### Decreased response to IL-2 in CD25^hi^ T cells inversely correlates with soluble IL-2RA levels in the serum of control subjects homozygous for the IL2RArs2104286 risk haplotype

**Figure 4:**
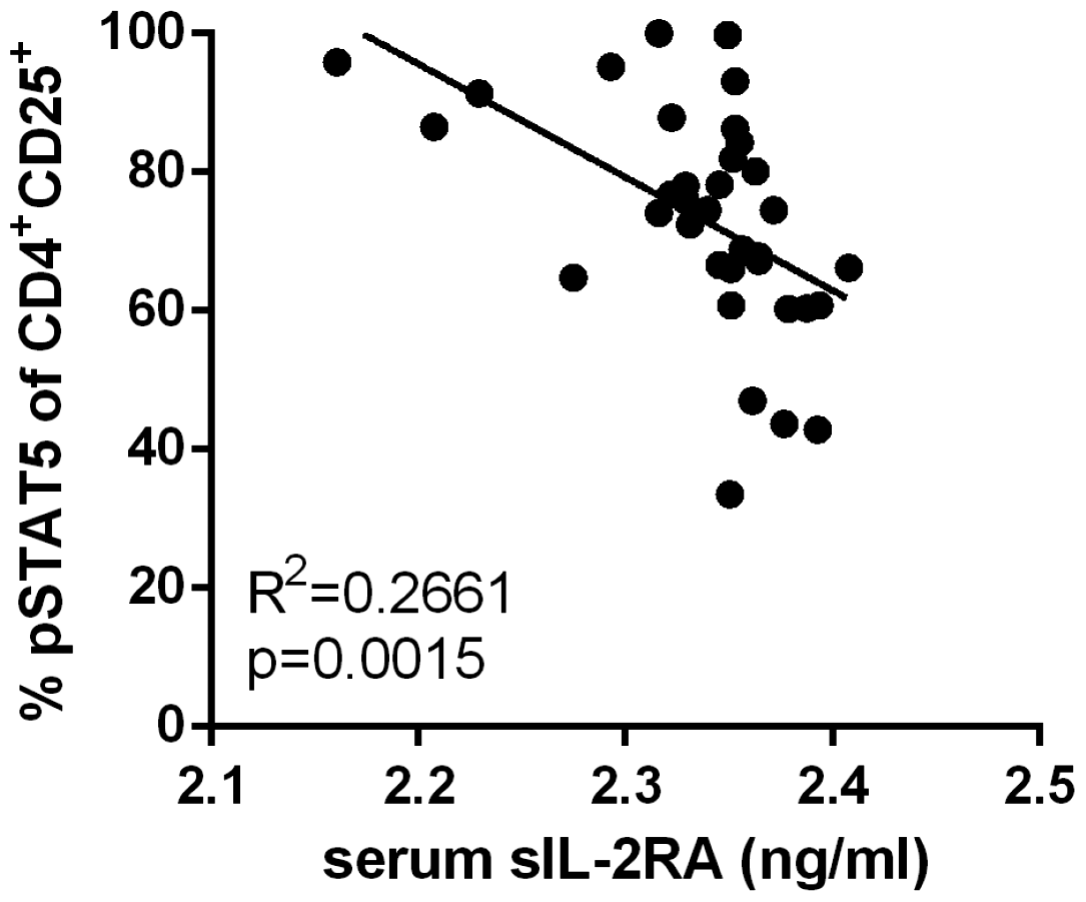
Increased serum sIL-2RA correlates with lower response to IL-2. PBMC and serum samples from the same blood draw were assayed for response to IL-2 as in Figure 1 and serum sIL-2RA using Luminex. All controls tested were Caucasian with no significant difference in age or gender between genotypes. Multivariable analysis was performed using linear regression and is shown in the Figure. To confirm these results we performed several additional tests. Random sampling of 80% of the data 1000 times resulted in a Pearson correlation of −0.516 and a p-value of 0.0015.Significance was also tested using a non-parametric correlation (Spearman test = −0.581, p-value of 0.0003) verifying that outliers are not the reason for statistical significance.

Previous studies have shown that increased sIL-2RA in the serum is associated with the *IL2RA*rs2104286 risk haplotype in healthy controls [16], [24], [31]. To better understand whether there is a relationship between response to IL-2 and the level of sIL-2RA shed from the cell surface, we correlated IL-2R signaling and sIL-2RA levels using serum and PBMC from the same blood draw of genotyped controls. When compared with the response to IL-2, we found an inverse correlation between serum sIL-2RA and response to IL-2 in the CD25^hi^ population (Figure 4). A mechanism which could explain the inverse relationship between sIL-2RA and pSTAT5 in response to IL-2 is the blockade of IL-2 binding to the IL-2R by sIL-2RA shed in the media. However, in our *in vitro* system this is unlikely since T cells are purified by ficoll gradient centrifugation and are subsequently frozen, but to further test this we washed cells prior to adding IL-2 (thereby eliminating the possible impact of soluble IL-2RA) or incubated overnight (allowing for accumulations of soluble IL-2RA). In both cases we found the response to IL-2 stayed the same as our initial measurement (data not shown). Thus, these data reveal an inverse relationship between response to IL-2 and sIL-2RA, but the mechanisms by which this alters IL-2 signaling are still unclear.

### PTPN2 and IL2RA independently contribute to reduced response to IL-2 in controls

**Figure 5:**
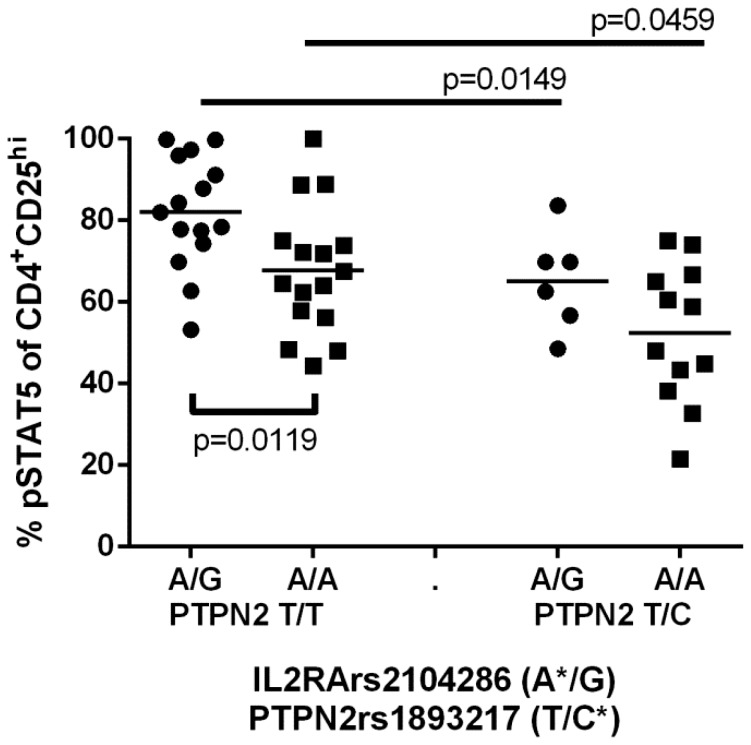
*IL2RA* and *PTPN2* genotype independently contribute to decreased IL-2R signaling in controls. PBMC were stained as in Figure 1. The frequency of pSTAT5 of CD25^hi^ T cells in control subjects were stratified by *PTPN2*rs1893217 and *IL2RA*rs2104286 haplotype. The mean pSTAT5 response is indicated for each group. Statistical significance between rs2104286 haplotypes within groups defined by PTPN2 genotype was determined using a Mann Whitney test. When analyzed as covariates in PLINK, age and gender did not significantly contribute to this phenotype (p = 0.4459 and p = 0.4954, respectively) and there was no evidence for interaction between *IL2RA* and *PTPN2* genotype (p = 0.7688). *denotes the risk haplotype determined by looking at variation at the rs21042856 allele while holding the rs12722495 allele constant at T/T and the rs11594656 allele constant at T/T.

We have previously shown that the rs1893217 SNP in *PTPN2*, a gene associated with T1D, RA and CD but not MS, correlates with decreased response to IL-2 in CD25^hi^ and memory CD25^lo^ cells [27]. To determine whether multiple genetic factors could contribute to the decreased response to IL-2 in CD25^hi^ T cells, we stratified control subjects by both the *PTPN2*rs1893217 genotype and the *IL2RA*rs2104286 haplotype and tested for interaction of these genotypes by linear regression using the PLINK toolset. A significant decrease in pSTAT5 was associated with the *IL2RA*rs2104286 risk haplotype group independent of *PTPN2*rs1893217 genotype using an additive model with age and gender as covariates (p = 0.028, Figure 5). Likewise, a significant decrease in IL-2 responsiveness was associated with *PTPN2*rs1893217 risk genotype independent of *IL2RA*rs2104286 haplotype (p = 0.0018), however there was no significant evidence for interaction of *IL2RA*rs2104286 and *PTPN2*rs1893217 genotypes (p = 0.7688). Thus, both the *IL2RA*rs2104286 risk haplotype and the *PTPN2*rs1893217 risk allele independently contribute to reduced IL-2R signaling when the risk genotypes are present together.

### PTPN2 and CD25 risk alleles are not the only factors contributing to decreased response to IL-2 in T1D and MS patients

**Figure 6:**
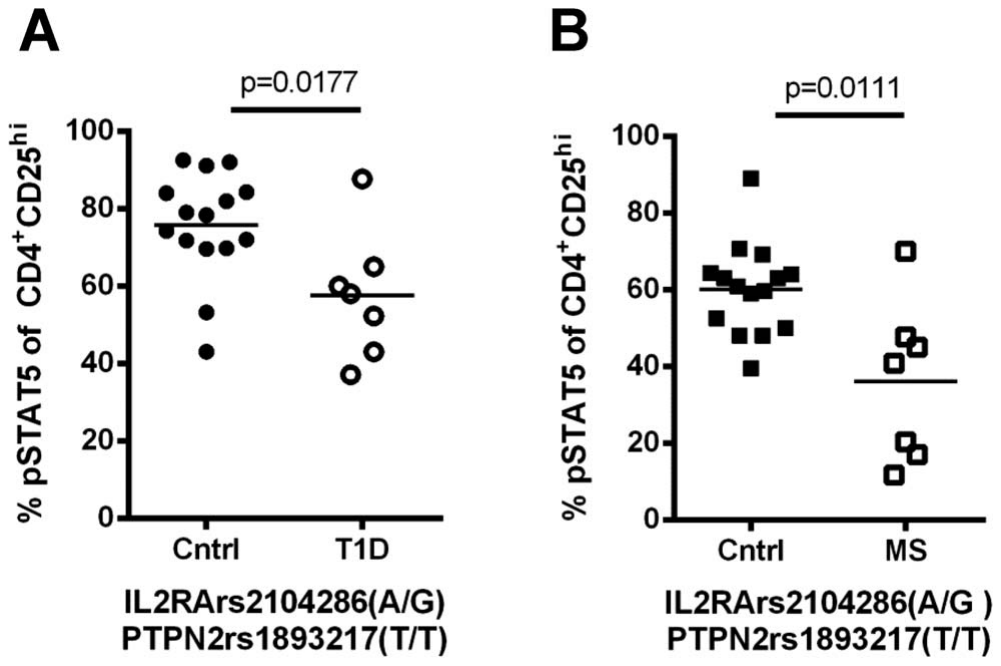
Factors beyond *IL2RA* and *PTPN2* variants contribute to reduced IL2R signaling in T1D and MS subjects. (A) T1D and (B) MS subjects carrying the protective *IL2RA*rs2104286 haplotype and the protective *PTPN2*rs1893217 SNP were compared to genotype, age and ethnicity matched controls. T1D subjects and matching controls were stimulated with 100 IU/ml IL-2 for 10min while MS samples and matching controls were stimulated with 25 IU/ml IL-2 for 20min. The mean pSTAT5 response is indicated for each group. Statistical significance was determined using a Mann Whitney test. *denotes the risk haplotype determined by looking at variation at the rs21042856 allele while holding the rs12722495 allele constant at T/T and the rs11594656 allele constant at T/T.

To determine whether the *IL2RA*rs2104286 risk haplotype and the *PTPN2*rs1893217 risk alleles together could explain the reduced response to IL-2 in T1D and MS patients relative to control subjects, we held the genotype at *IL2RA* and *PTPN2* constant for the protective alleles and compared responses in disease patients to age and ethnicity matched controls (Figure 6A and B). As in previous analyses, all subjects carried the common risk allele at *IL2RA*rs12722495, a SNP previously shown to display decrease CD25 expression and IL2R signaling [25], [32]. Even when accounting for *IL2RA* and *PTPN2* genotype, we still find a significant decrease in the response to IL-2 in the CD25^hi^ T cell populations of T1D and MS subjects. In addition, we used the model of y =  α + β_1_x_IL2RA_ +β _2_x_PTPN2_ +β _3_x_IL2RA*PTPN2_ + ε to assess the interaction of each variant in the control, T1D and MS cohorts shown in Figure 1. Although certain combinations of SNP interactions could not be tested based on our sample size, we found no interaction between variants that were present in the disease cohorts (controls p = 0.32; T1D p = 0.89; MS p = 0.19 by ANOVA). Moreover, we found no significant impact on percentage of pSTAT5 when disease status (p = 0.98) and treatment (0.40) were included as variables in the analysis for the MS cohort. Thus, other factors in addition to *IL2RA* and *PTPN2* genotype likely contribute to reduced response to IL-2 in these two autoimmune diseases.

## Discussion

Since IL-2 plays a key role in maintaining tolerance, it follows that patients with autoimmune diseases may display defects in the IL-2/IL-2R signaling pathway. Here, we extend our previous finding that response to IL-2 is reduced in CD4^+^ T cells of T1D subjects [11] and find a similar defect in CD4^+^CD25^hi^ T cells of MS patients. Interestingly, this IL-2R signaling defect was not observed in our cohort of SLE patients. However, impaired production of IL-2 has been described in SLE [33]. Together, these results support the hypothesis that defects in the IL-2/IL-2R pathway are present in autoimmunity. We found that the *IL2RA*rs2104286 haplotype that is associated with T1D and MS, but not SLE, was associated with reduced IL-2R signaling in CD4^+^CD25^hi^ T cells of controls. In addition, we found that the risk alleles at *IL2RA* and *PTPN2,* a second IL-2R signaling pathway gene, could act independently to decrease IL-2 responsiveness in control subjects. However, genetic variation at *IL2RA*rs2104286 and *PTPN2* does not account for all of the decreased signaling observed in patients. Thus, multiple factors including *IL2RA* genetic susceptibility contribute to reduced IL-2R signaling in T1D and MS patients.

Despite the similarities in the response to IL-2 in T1D and MS, there remain disease-specific differences. In this study, we found decreased signaling in response to IL-2 in CD25^hi^ T cells of both T1D and MS patients but decreased response in CD4^+^CD25^lo^ T cells of only T1D patients. Moreover, this decrease did not correlate with therapy or disease status (Figure S3). Thus, IL-2R signaling defects may be more pervasive in T1D. This is consistent with previous studies demonstrating decreased response to IL-2 and IL-15 in T1D patients [11] and controls carrying the T1D-associated *PTPN2*rs1893217 risk allele [27], a gene not associated with MS.

To date, cellular phenotypes linked to the *IL2RA* locus have been best defined for the T1D-associated *IL2RA*rs12722495 haplotype. These occur in both CD25^hi^ T cells and memory Teff cells and include decreased IL2RA RNA and CD25 surface expression in Treg (confirmed in Figure S2) and memory Teff [25], [32], [34], increased frequency of CD25^+^ naive T cells [25], decreased response to IL-2 [32] and increased serum sIL-2RA [16], [24]. In contrast, the *IL2RA*rs2104286 risk haplotype, associated with both T1D and MS, only shares a subset of these phenotypes, including increased frequency of CD25^+^ naïve T cells and increased serum sIL-2RA. In this study we did not observe decreased expression of CD25 on memory T cells with the rs2104286 risk haplotype, but we did detect increased CD25 surface expression on naïve Treg, a phenotype that was mirrored in both T1D and MS patients.

The molecular mechanisms underlying the phenotypes associated with *IL2RA* risk variants appear to be at least two-fold, involving transcriptional regulation of the *IL2RA* locus and production of sIL-2RA. All associated SNPs in the *IL2RA* gene to date are located in the promoter region or intron 1, and differential allelic expression of IL2RA transcripts has been reported in HapMap lymphoblastoid cell lines and T1D cases/parents [34]. However, the allele-specific expression was not associated with rs12722495, rs2104286, or rs11594656, but rather other SNPs in intron 1. Subsequent analysis of allele-specific transcription factor binding to SNPs in the *IL2RA* locus revealed that at least four *IL2RA* SNPs demonstrate differential binding [35]. This result taken together with the low R^2^ values for LD between SNPS in *IL2RA*, suggests that CD25 expression is the combined result of allele-specific transcription factor binding to the haplotype of alleles across the *IL2RA* promoter and intron 1in any particular individual, and may explain some of the genetic and phenotypic heterogeneity observed with this locus. Added to this composite expression picture are cell extrinsic factors, such as the production of matrix metalloproteases like MMP-9 which can cleave IL-2RA from the cell surface [36] and glycosylation of cytokine receptors that further impacts stability and function of the receptor [37]. However, production of sIL-2RA also seems to have a genetic component because it is associated with *IL2RA* SNPs in control subjects, including rs2104286 as we observed here, and is stable over time [16], [31]. The molecular basis for this genetic association is not clear, since no transcript encoding a sIL2RA isoform has been detected to date.

While increased levels of serum sIL-2RA are present in several autoimmune and inflammatory states and are associated with *IL2RA* genotype [16], [26], [31], the functional consequences of increased sIL-2RA are not well understood. Here, we find that increased sIL-2RA correlates with decreased response to IL-2. Taken together with others’ findings suggesting that sIL-2RA reduces IL-2 availability both *in vitro* and *in vivo* [1], [31], [38], one may conclude that sIL-2RA is functioning as a decoy thereby reducing the amount of IL-2 capable of binding the surface IL-2R. Yet, subjects with high sIL-2RA still displayed reduced pSTAT5 when cells were washed (data not shown), thereby eliminating the likelihood of sIL-2RA functioning as a decoy under these conditions.

To summarize our results and those of others, it appears that multiple mechanisms can cause reduced IL-2 signaling, contributing to autoimmune susceptibility and pathogenesis, as detailed in Table S2. Given the importance of Treg in tolerance and known defects in Treg of T1D and MS subjects [39], it is likely that decreased stability and function of Treg is a major mechanism by which *IL2RA* variants contribute risk in associated autoimmune diseases. However, other cell subsets may also be involved, particularly in T1D. Decreased response to IL-2 in Teff populations may cause impaired activation induced cell death leading to increased frequency of autoreactive Teff cells. Additionally, reduced IL-2 responsiveness has the potential to decrease induction of adaptive Treg while increasing Th17 generation [40]. Lastly, increased CD25 expression on naïve Treg (Figure 3) or increased frequency of CD25^+^ naive T cells may enhance the homeostatic proliferation of these naïve T cell subsets as suggested in a recent study [25], [41]. Whether this leads to senescence, reduced stability or alterations in the repertoire are currently under investigation, along with other work to more decisively link phenotypes to mechanisms of disease.

## Supporting Information

Figure S1 **Decreased response to IL-2 in CD4^+^CD25^+^ T cells correlates with** ***IL2RA*** **rs2104286.** PBMC from the GAP registry (see materials and methods) were thawed and stained as in Figure 1. The frequency of pSTAT5^+^ cells of CD25^hi^ and CD25^lo^ gated cells was determined by comparing media to IL-2 stimulated genotyped control samples. Subjects with *IL2RA*rs2104286 genotypes G/G (▴) and A/A (▪) are differentiated by symbol shapes. Statistical significance was determined using a Mann Whitney test. *denotes the risk haplotype determined by looking at variation at the rs21042856 allele while holding the rs12722495 allele constant at T/T and the rs11594656 allele constant at T/T.
(PPTX)

Figure S2 **Decreased CD25 on memory Treg when stratified by** ***IL2RA*** **rs12722495.** PBMC were thawed and stained for CD4, CD25, FOXP3, helios and CD45RA as described in materials and methods and gated as shown in Figure 3A.. CD25 MFI was measured on CD45RA^+^ naïve and CD45RA^−^ memory Treg populations of controls with *IL2RA*rs2104286 genotypes: G/G (▴), A/G (○) and A/A (▪). Statistical significance for individual pairings using a Mann-Whitney test are shown.
(PPTX)

Figure S3 **Therapy and disease status do not correlate with response to IL-2 in CD25^hi^ cells of the T1D, MS or SLE cohorts.** Data from Figure 1A were analyzed against clinical parameters for the **(A)** T1D, **(B-C)** MS and **(D)** SLE cohorts. Statistical significance was determined using linear regression in (A, C) and a Mann Whitney test in (B). The mean pSTAT5 response is indicated for each group in (B, D). *IL2RA*rs2104286 genotype is noted by solid black (A/A), solid grey (A/G) and open (G/G) symbols where measured in the T1D and MS cohorts.
(PPTX)

Table S1 Results of Mann-Whitney association testing and 95% confidence intervals for all comparisons in the figures.
(XLSX)

Table S2 Summary of IL-2/IL-2R signaling phenotypes and suggested mechanisms contributing to autoimmunity.
(DOCX)
