# Supplementary material for: Multiple Autoimmune-Associated Variants Confer Decreased IL-2R Signaling in CD4+CD25hi T Cells of Type 1 Diabetic and Multiple Sclerosis Patients
Source: PLoS One. 2013 Dec 23;8(12):e83811. doi: 10.1371/journal.pone.0083811 (PMC3871703; doi:10.1371/journal.pone.0083811)
Supplement: Table S2 — Summary of IL-2/IL-2R signaling phenotypes and suggested mechanisms contributing to autoimmunity. (DOCX) [file pone.0083811.s005.docx]

Table S2: Summary of IL-2/IL-2R signaling phenotypes and suggested mechanisms contributing to autoimmunity

| **Phenotype** | **Cohort^a^** | **Hypothesized role in autoimmunity** | **ref** |
| --- | --- | --- | --- |
| ↓ pSTAT5 to | ***IL2RA*rs12722495 risk control** | Reduced stability and function of | 34 |
| IL-2 in Treg | *IL2RA*rs2104286 risk control | IL-2 dependent Treg | Fig 1 |
|  | *PTPN2*rs1893217 risk control |  | Fig 2 |
|  | T1D |  | Fig 6 |
|  | MS |  |  |
| ↓ pSTAT5 to | ***PTPN2*rs1893217 risk control** | Reduced activation induced cell of | 25 |
| IL-2 in Teff | *IL2RA*rs12722495 risk control | death and IL-2 dependent functions | Fig 1 |
|  | T1D | Teff | Fig 6 |
| ↓ CD25 RNA and protein | *IL2RA*rs12722495 risk control | Reduced Treg stability and function and reduced Teff activation induced cell death | 25, 35 |
| ↑ serum | *IL2RArs11595646* | Decoy for IL-2 leading to reduced | 16 |
| sIL2RA | *IL2RA*rs2104286 risk control | IL-2 response in Treg and Teff and | 32 |
|  | T1D | biomarker of increased proliferation | Fig 4 |
|  | MS |  |  |
| ↑ % CD25^+^ | *IL2RA*rs2104286 risk control | Increased proliferation of naïve | 42 |
| naïve T cells | *IL2RA*rs12722495 risk control | cells leading to senescence and |  |
|  |  | changes in the naïve repertoire |  |
| ↑ CD25 | *IL2RA*rs2104286 risk control | Increased proliferation of naïve | Fig 3 |
| expression on | T1D | cells leading to senescence and |  |
| naïve Treg | MS | changes in the naïve repertoire |  |

^a^Cohorts in bold have the strongest effect on the phenotype
